# Adverse Drug Reactions of Cardiovascular Classes of Medicines—Data for Bulgarian Population

**DOI:** 10.3390/biomedicines12102163

**Published:** 2024-09-24

**Authors:** Zornitsa Mitkova, Anita Dimova, Guenka Petrova, Maria Dimitrova

**Affiliations:** Faculty of Pharmacy, Medical University-Sofia, 1000 Sofia, Bulgaria; anidimova24@gmail.com (A.D.); gpetrova@pharmfac.mu-sofia.bg (G.P.); mdimitrova@pharmfac.mu-sofia.bg (M.D.)

**Keywords:** drug-related side effects and adverse reactions, antihyperlipidemic medicines, angiotensin-converting enzyme(ACE)inhibitors, sartans

## Abstract

**Objective:** Hypertensionis one of the most common chronic diseases, affecting more than 20% of the population. The side effects experienced due to antihypertensive medications, such as tiredness, muscle pain, and insomnia, are often a significant predictor of poor adherence to therapy. The goal of the current study is to present the frequency, type, seriousness, and severity of adverse drug reactions reported to the BDA via Individual Case Safety Reports (ICSRs) and following differentiation of messages found in more than one patient. **Methods:** We conducted a retrospective analysis of the reported adverse drug reactions (ADRs) reported in the Bulgarian Drug Agency database after treatment with antihyperlipidemic medicines, angiotensin-converting enzyme (ACE) inhibitors, and sartans for the period 2017–2021. Each ICSR form was observed, and data for suspected medicine and type of adverse reaction was analyzed. **Results:** The total number of processed notifications for adverse drug reactions (ADRs) included in the database is 142. The highest number of ADRs was reported for ARB (58), followed by antihyperlipidemic medicines (55) and ACE inhibitors (29). Most of the assessed adverse events experienced by more than one patient fall into the probable and related categories based on the Global Introspection method classification. Therefore, they have been investigated and are consistent with exposure in the population. **Conclusions:** Cardiovascular medicines from the groups of ACE inhibitors, sartans, and statins have a high share of reported ADRs in the BDA system. Some of them are severe and need further investigation.

## 1. Introduction

Cardiovascular diseases (CVDs) are the leading cause of co-morbidity and polypharmacy due to their complex nature. Hypertension is one of the most common chronic CVDs, affecting more than 20% of the population. Hypertension prevalence in Bulgaria is 48%, compared with a global average of 33%. Out of 2.4 million hypertensive patients aged 30–79 in Bulgaria, only 28% of females and 18% of males successfully managed the disease. Not only morbidity but also mortality is high. Deaths due to cardiovascular disease accounted for 68,900 in 2019 [1].

National programs for early hypertension diagnosis among the aging population led to the detection of more co-morbid conditions compared with those 20 years ago. Extended life expectancy and longer years of life with chronic conditions have increased the number of people who needed long-term therapy worldwide. Statistical data confirms that over 70% of people above 65 years of age have cardiovascular co-morbidities [2]. CVD treatment often requires more than two medications, thus enhancing the polypharmacy in this group and increasing the risk of adverse events (fall injury, heart failure, and BP exacerbation) [3]. In addition to a higher risk of side effects, drug interactions are also foreseeable. About 13% of elderly people are taking over 10 medicines, thus increasing the risk and exposure to adverse events [4]. One-third of the chronic patients had concerns for the necessity of their medicines [5]. A higher number of prescribed medicines correlated with observed adverse events such as hospitalizations, mortality, side effects, and medicine interactions [6]. Some of them might be life-threatening, for example, bleeding and thrombotic events in patients with atrial fibrillation (AF) on polypharmacy (or complexity of major bleeding, ischemic stroke, and mortality) [7].

The increased frequency of hospitalizations and complications is the main reason for significant public expenditure [8]. The side effects experienced due to antihypertensive medications, such as tiredness, muscle pain, and insomnia, are often a significant predictor of poor adherence to therapy [9]. The risk of medication non-adherence depends on some therapy-related factors such as the number of prescribed medicines, prescribed drug classes, and their safety profile [10]. Single-pill combination (SPC) therapy in patients suffering from hypertension and dyslipidemia is associated with systolic blood pressure reduction, reduced emergency department visits, and better clinical outcomes compared with free combination therapy (FCT) [11].

There is evidence that adverse drug reactions (ADRs) are underreported [12]. To overcome this challenge, pharmaceutical legislation introduced the possibility of reporting ADRs by patients and all health care professionals, in addition to companies’ obligations to provide evidence for the safety of their products [13].

This study aims to systematize ADRs for ACE inhibitors, sartans, and antihyperlipidemic medicines reported to the Bulgarian Drug Agency (BDA) via Individual Case Safety Reports (ICSRs) according to their frequency, type, seriousness, and severity. Repeating ADRs appearing in more than one patient were also evaluated.

## 2. Materials and Methods

We conducted a retrospective, descriptive analysis of the ADRs reported to the Bulgarian Drug Agency database for the period 2017–2021. Information from every ICSR was extracted and analyzed according to:

- Suspected medicines (international nonproprietary name (INN) and therapeutic indication),as well as concomitant therapy;
- Characteristics of the reported adverse event as evaluated by the BDA: seriousness; expectancy; severity; type of report and reporting (spontaneous or nonspontaneous); adverse event occurred (MedDRA) related to the suspected drug, and reporter-physicians, patients, and other healthcare professionals (including experts from pharmaceutical companies, pharmacists, etc.).

Additionally, subgroup analysis was performed for events that occurred in more than one patient. They were analyzed according to their frequency and causality. The causal relationship of suspected ADRs was evaluated using the Global Introspection method [14] from a healthcare professional’s point of view. The company or primary source reporters’ assessment was provided in cases of missing evaluation from the health care professionals.

## 3. Results

The total number of processed notifications for adverse drug reactions (ADRs) included in the database during 2017–2021 is 142. Out of these, reports for antihyperlipidemic medicines were 55, of which 20 were classified as serious ADRs, 28 as expected, and 42 as spontaneous. Rosuvastatin is the product with the highest number of ADR reports, accounting for 45.45% of the total number of reported notifications of antilipidemics, with 29.09% of them are serious.

In the group of angiotensin II receptor blockers (sartans), 58 reports were found, with 46 serious, 16 expected, and 57 spontaneous ADRs. The highest number of reports was submitted for valsartan (48.28%), and the number of serious ADRs (39.66%) was also the highest. In total, 29 reports for ACE inhibitors were submitted to the BDA. Of these, 13 were reported serious, 26 expected, and 25 spontaneous ADRs. The highest number of reports and the most serious ADRs were for ramipril (41.38% of which 20.6% were serious).

Table 1 presents a summary of submitted notifications for each INN.

The highest number of notifications was received for valsartan—19.72%, followed by rosuvastatin—17.61% and evolocumab—12.68%. The total number of serious adverse events is 79; of these 40% are those of valsartan, 29% of rosuvastatin, and 17% of olmesartan (10)—Table 1.

The adverse drug reactions occurring in more than one patient, along with the available data for causality included in ICSR, are presented on Table 2. The categories are based on an assessment of causality provided by health professionals using the Global Introspection scaling.

ADRs occurring in more than one patient were observed for 10 out of 14 INNs included in the observation. In the group of statins, atorvastatin, rosuvastatin and evolocumab, ADRs affected between 2 to 5 persons, with the highest number being 23 patients for evolocumab. Acute myocardial infarction, acute coronary syndrome, and angina pectoris were the most frequently reported. Due to their potentially life-threatening nature, there is a need for additional examination of product safety profiles.

Within the sartans group, identical ADR appearing in more than one person were reported for irbesartan, olmesartan, telmisartan, and valsartan. Serious ADRs reported frequently in more than one patient manifested as cutaneous malignancies (melanoma in 22 people), squamous cell carcinoma (n = 4), as well as cases of prostate carcinoma (n = 2). Such a high number is impressive in comparison with other sartan-related reports. These ADRs appear within 2 to 5 years after the beginning of sartan therapy and should be carefully examined, especially for valsartan. ADRs were reported in 6 out of 7 patients during treatment with irbesartan, 12 out of 13 treated with olmesartan, 2 out of 7 for telmisartan, and 22 out of 28 during therapy with valsartan. These adverse drug reactions are unexpected (not mentioned in the summary of product characteristics), possible, and have a suspected causal relationship, especially when more than one sartan is taken.

Within the ACE inhibitor group, adverse drug reactions occurring in more than one patient have been reported for ramipril, lisinopril, and perindopril. The most severe reactions are heart and valvular failure, cardiotoxicity (n = 2), pulmonary hypertension (n = 2), and ventricular tachycardia (n = 3). Hypo- and hyperglycemia have also been reported in 4 diabetics.

Patients from the 45-65 years of age group prevail in reported ADRs. This is probably due to the fact that this age group is the largest group with chronic diseases, polymorbidities, and polypharmacy, as well as being more frequent users of medicines. The correlation between the number of ADRs and other patient characteristics was not explored because it was not within the scope of the analysis.

**Figure 1:**
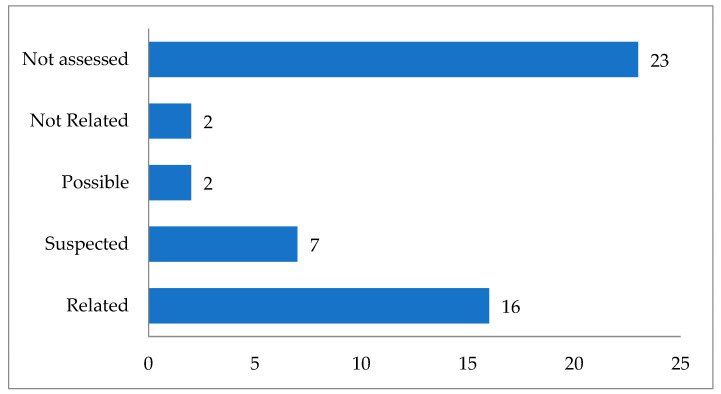
Distribution of causality categories of reported ADR in more than one patient.

Most of the ADRs experienced by more than one patient fall into the related and suspected categories, with 16 and 7 respectively, indicating that they have been investigated and are consistent with exposure to the INN (Figure 1). 

Despite the missing category ‘suspected’ in the Global Introspection classification, we found that the side effects of ramipril and valsartan are assessed as suspected in provided ICSR. Therefore, they are presented as suspected in the figure above.

## 4. Discussion

In this study, we evaluated ADRs reported to the BDA for three classes of CV medicines. We found that there has been an increase in reporting, and the physicians are the most active reporters in the system. It is also evident that many of the reported ADRs occur in more than one patient, and many of them are severe.

Polypharmacy is one of the most important factors for the occurrence of ADRs, and patients with CVD are among those most frequently affected [15]. Factors that contribute to the development of adverse reactions may be related to the medicinal product, to the disease, or to the patient. Age, gender, and disease progression are patient-related factors that contribute significantly to the development of adverse reactions, especially among patients with CVD [16]. We have not yet analyzed these data and will further deepen our analysis.

Lesar et al. found that ADRs for CV medicines were 2.4 times higher compared to other products [17]. Our results confirm such a conclusion, as most of the reported ADRs to the BDA were for CV medicines.

A combination of some types of antidiabetics with antihypertensive medicines could result in hypoglycemia in patients with diabetes mellitus [18]. Therefore, cardiovascular medicines, which are among the most commonly prescribed in healthcare settings, can be precursors for medication errors and should be monitored for associated side effects [19]. We do have some suspicious results regarding possible drug interactions that need to be further investigated.

The Danish study found that enalapril and ramipril were the most frequently prescribed, with 40.3% and 42.6% of the market share of ACE- inhibitors. However, 54.7% of adverse events were associated with enalapril, while only 14.2% concerned ramipril [20]. As it was examined in Bulgaria, ramipril utilization was higher than that of enalapril (19.61 vs. 12.12 reference DDD/1000 in h/day in 2019) and continues to rise in recent years [21]. The number of reported ADRs for ramipril is higher (n = 12 or 41.38%), followed by perindopril (37.9%), and enalapril (10.34%). The most common ADRs for ACE inhibitors are cough, hypotension, hyperkaliemia, and acute renal failure. Similar to our results, majority of ADRs were probable and assessed as moderately severe [22].

Evolocumab often leads to flu-like symptoms and general malaise, which is expected due to its affiliation with the monoclonal antibody group. It is a relatively new hypolipidemic drug and still requires additional safety studies. Similar to our findings, the American Heart Association published data on side effects in more than one patient exposed to statins [23], which increased diabetes risk, risk of hemorrhagic stroke, and could also lead to back pain. The interesting fact is that muscle pain and weakness are not reported in Bulgaria.

Recent studies also discussed cancer risk during sartan treatment, but the causal link remains uncertain. Moreover, a population-based 10-year study provides evidence that an increase in cancer risk is unlikely [24]. Nitrosamine impurities should also be examined to exclude or confirm potential relationship [25]. Nitrosamines have a proven carcinogenic potential and can cause heterogeneous neoplasms, which means there could be possible melanoma triggers [26,27].

The range of patient-reported ADRs is from 57.83% to 14.37% in five selected Western EU countries, whereas less than 1% of patients in Asia reported such. The developed feedback system, supported by patient organizations, and increasing citizens’ awareness are among the leading factors for the higher levels of ADRs reported by the patients [28]. Our study showed that physicians are the most active group reporting adverse events. Reports from patients account for 16.2%, which is the low range for EU countries. BDA reports showed a slight increase in recent years [29]. The necessity of patient education and the popularization of the BDA platform, which allowed direct reporting, is obvious.

This is the first descriptive study in Bulgaria focusing on reported adverse drug reactions in cardiology patients using the BDA database. The limitation of our study is that it is a descriptive analysis of the reporting, and correlation between patient characteristics and ADRs was not analyzed. We are planning further studies to explore in more detail the population risk for various ADRs and patient characteristics. Concomitant therapy, co-morbidities, and the correlation between patients’ personal characteristics and disease progression are also subjects of further studies. With this study, we aimed only to present the reporting activities and ADR distribution according to the drug agency’s grouping.

## 5. Conclusions

Reporting of ADRs in cardiology provides information regarding patient risks and aids physicians in prescribing. This study found that the reporting of ARDs in cardiology is increasing. ACE inhibitors, sartans, and statins are the therapeutic groups for which most ADRs were reported in the drug agency system. A total of 79 out of 142 notifications for ADRs were serious, which can have a significant impact on the healthcare system. ADRs related to sartans, which are among the most frequently used cardiovascular groups, prevail in reports. In more than one patient, severe ADRs related to sartans, such as carcinoma and malignancies, were reported that needs to be analyzed further. Lots of ADRs appearing in more than one person were unexpected, which should increase attention from companies and regulators.

## Figures and Tables

**Table 1:** Distribution of ADRs by number, severity, expectancy, reporting type, and person.

INN	Number (%) of Reports	Number (%) of Serious ADRs	Number (%) of Expected ADRs	Number (%) of Spontaneous Reports	Number of Reports Submitted by		
					Physician	Patient	Other Healthcare Professional
Atorvastatin	7 (12.73)	2 (3.64)	6 (10.91)	7 (12.73)	5	-	2
Simvastatin	3 (5.45)	-	3 (5.45)	3 (5.45)	-	1	2
Rosuvastatin	25 (45.45)	16 (29.09)	5 (9.09)	25 (45.45)	20	3	2
Ezetimibe	2 (3.64)	-	1 (1.82)	2 (3.64)	-	1	1
Evolocumab	18 (32.73)	2 (3.64)	13 (23.64)	5 (9.09)	11	5	2
Candesartan	3 (5.17)	2 (3.45)	1 (1.72)	3 (5.17)	1	2	
Irbesartan	7 (12.07)	7 (12.07)	-	7 (12.07)	5	-	2
Olmesartan	13 (22.41)	10 (17.24)	7 (12.07)	13 (22.41)	6	2	5
Telmisartan	7 (12.07)	4 (6.90)	4 (6.9)	7 (12.07)	3	2	2
Valsartan	28 (48.28)	23 (39.66)	4 (6.9)	27 (46.55)	20	2	6
Ramipril	12 (41.38)	6 (20.69)	11 (37.93)	12 (41.38)	3	2	7
Enalapril	3 (10.34)	2 (6.90)	3 (10.34)	3 (10.34)	2	1	-
Lisinopril	3 (10.34)	1 (3.45)	3 (10.34)	3 (10.34)	1	-	2
Perindopril	11 (37.93)	4 (13.79)	9 (31.03)	7 (24.14)	3	2	6

The percentages calculated in the table are based on the total number of reports for each pharmacotherapeutic group (55 for antihyperlipidemics, 58 for sartans, and 29 for ACE inhibitors).

**Table 2:** ADRs occurring in more than one patient and including causality assessment in ICSR.

INN	ADR in More than One Patient	Causality Assessment According to **Global Introspection**	Number of Patients (% from Total Number of INN Notifications) *	Patient’s Age, Years
Atorvastatin	Increased glucose level	Related, Global Introspection, Health Care professional	2 (28.57)	45–65
	Hypoglycaemia	Related, Global Introspection, Health Care professional	2 (28.57)	45–65
	Dry mouth	Related, Global Introspection, Health Care professional	2 (28.57)	45–65
	Polydipsia	Related, Global Introspection, Health Care professional	2 (28.57)	45–65
	IncreasedHbA1C	Related, Global Introspection, Health Care professional	2 (28.57)	45–65
	Pollakiuria	Related, Global Introspection, Health Care professional	2 (28.57)	45–65
Rosuvastatin	Chest pain	not assessed	4 (16.00)	45–65
	Fatigue	not assessed	3 (12.00)	45–65
	Hypertension	not assessed	3 (12.00)	45–65
	Acute myocardial infarction	not assessed	5 (20.00)	45–65 and 25–44 (2 patients)
	Acute coronary syndrome	not assessed	3 (12.00)	45–65
	Unstable angina	not assessed	4 (16.00)	Over 65
Evolocumab	Influenza like illness	not assessed	4 (22.22)	45–65 (2 patients), over 65 (2 patients)
	Back pain	not assessed	3 (16.67)	45–65
	Limb pain	not assessed	3 (16.67)	45–65
	Vomiting	not assessed	2 (11.11)	45–65
	Myalgia	not assessed	3 (16.67)	45–65, over 65 (2 patients)
	Rhinorhoea	not assessed	3 (16.67)	45–65, over 65 (1 patient)
	Positive test for COVID-19	not assessed	2 (11.11)	45–65
	Hematoma at the injection site	not assessed	3 (16.67)	45–65
Irbesartan	Malignant melanoma	Related, Global Introspection, primary source reporter	6 (85.71)	45–65 (1 patient), over 65
	Prostate Carcinoma Possible	not assessed	2 (28.57)	over 65
Olmesartan	Malignant melanoma	not assessed	8 (61.54)	45–65 (1 patient), over 65
	Squamous cell carcinoma	not assessed	4 (30.77)	over 65
	Headache	Related, Global Introspection Reporter	2 (15.38)	over 65
Telmisartan	Malignant melanoma	Possible, Global introspection, Author	2 (28.57)	over 65
	Hypertension	not assessed	2 (28.57)	45–65
	Fatigue	not assessed	2 (28.57)	45–65
	Chest pain	not assessed	2 (28.57)	45–65
Valsartan	Malignant melanoma	Possible, Global Introspection, Author	22 (78.57)	45–65 (7 patients), over 65
	Squamous cell carcinoma	not assessed	4 (14.29)	over 65
	Pruritus	Suspected, Global Introspection, primary source reporter	3 (10.71)	45–65 (1 patient), over 65
Ramipril	Pulmonary hypertension	Suspected, Global Introspection, primary source reporter	2 (16.67)	45–65
	Chest pain	Not assessed	3 (25.00)	45–65
	Fatigue	Suspected, Global Introspection, primary source reporter	2 (16.67)	45–65
	Cough	Related, Global Introspection, Health Care professional	3 (25.00)	45–65, 25–44 (1 patient)
	Joint swelling	Not Related, Global Introspection, Health Care professional	3 (25.00)	45–65, 25–44 (1 patient)
	Heart failure	Suspected, Global Introspection, primary source reporter	2 (16.67)	45–65
	Mitral valve incompetence	Suspected, Global Introspection, primary source reporter	2 (16.67)	45–65
	Tricuspid valve insufficiency	Not Related, Global Introspection, Health Care professional	2 (16.67)	45–65
	Cardiotoxicity	Suspected, Global Introspection, primary source reporter	2 (16.67)	45–65
	Dyspnoea	Suspected, Global Introspection, primary source reporter	2 (16.67)	45–65
Lisinopril	Hyperglycaemia	Related, Global Introspection, Health Care professional	2 (66.67	45–65
	Hypoglycaemia	Related, Global Introspection, Health Care professional	2 (66.67)	45–65
	Dry mouth	Related, Global Introspection, Health Care professional	2 (66.67)	45–65
	Polydipsia	Related, Global Introspection, Health Care professional	2 (66.67)	45–65
	Polyuria	Related, Global Introspection, Health Care professional	2 (66.67)	45–65
	Increased HbA1C levels	Related, Global Introspection, Health Care professional	2 (66.67)	45–65
Perindopril	Ventricular tachycardia	not assessed	3 (27.27)	25–44, over 65 (1 patient)
	Heart failure	Related, Global Introspection, Health Care professional	2 (18.18)	25–44

* The percentage is calculated from the total number of reports for each INN, as presented in the first column of Table 1.

## Data Availability

Data are Available after a reasonable request from the authors.
